# "Nutritional and chemopreventive anti-cancer agents up-regulate expression of p27Kip1, a cyclin-dependent kinase inhibitor, in mouse JB6 epidermal and human MCF7, MDA-MB-321 and AU565 breast cancer cells"

**DOI:** 10.1186/1475-2867-6-20

**Published:** 2006-08-09

**Authors:** Isao Eto

**Affiliations:** 1 Department of Nutrition Sciences, University of Alabama at Birmingham, Birmingham, Alabama, USA

## Abstract

**Background:**

p27(Kip1) is a cyclin-dependent kinase inhibitor. When up-regulated, p27 inhibits G1-to-S phase transition of the cell cycle. This report addresses the question of whether various nutritional and chemopreventive anti-cancer agents up-regulate the expression of p27 in preneoplastic and neoplastic cells.

**Results:**

Experimental evidence presented in the first half of this report shows that these agents fairly faithfully up-regulate expression of p27 in mouse epidermal (JB6) and human breast cancer (MCF7, MDA-MB-321, and AU565) cells. Up-regulation appears to be specific to p27 because expression of cyclin D1, E, and A, and p21Cip1/Waf1 was not modulated by these agents. Up-regulation of the expression of p27 is likely due to the activation of translation rather than transcription of p27 because (a) up-regulation is mediated by the 5'-untranslated region (-575) of the *p27 *gene and (b) the antibiotic actinomycin D, an inhibitor of transcription, did not attenuate the up-regulation of p27. This latter finding is likely to preclude the existence of cryptic transcription factor binding site(s) in the 5'-untranslated region of p27 gene. The experimental evidence, presented in the second half of this report, was obtained using the 5'-untranslated region (-575) of *p27 *gene. The evidence suggests that cancer preventive agents up-regulate expression of p27 by at least four different molecular signaling pathways: (a) Caloric restriction is likely to up-regulate p27 expression via 5'-AMP-activated protein kinase (AMPK; a metabolic energy sensor or cellular fuel gauge), tuberous sclerosis complex (TSC), and mammalian target of rapamycin (mTOR). Amino acid deficiencies also up-regulate the expression of p27 using some components of this pathway. (b) 4-Hydroxytamoxifen (but not tamoxifen), genistein (but not genistin), daidzein, and probably other nutritional and chemopreventive anti-cancer agents could up-regulate expression of p27 via receptor protein tyrosine kinases (RPTKs), phosphoinositide 3-kinase (PI3K), phosphoinosite-dependent kinase (PDK), Akt/PKB and mTOR. (c) Expression of p27 could also be up-regulated via RPTKs followed by MAPKs – MEK, ERK and p38MAPK – and probably MNK. Finally, (d) global hypomethylation of 5'-m^7^G cap of mRNAs could also up-regulate expression of p27.

**Conclusion:**

Based on these findings, we conclude that various nutritional and chemopreventive anti-cancer agents up-regulate expression of p27 in (pre)neoplastic cells.

## Background

The cyclin-dependent kinase (CDK) inhibitor p27(Kip1) is a key cell-cycle regulator of G1-to-S phase transition [1]. Transcriptional and translational control, sequestration in cyclin D1 complexes and localization all regulate p27 in G1 phase.

Preliminary studies using either *N*-methyl-*N*-nitrosourea (MNU)-induced rat breast cancer model or human breast cancer cell lines *in vitro *had suggested, but not proved, that nutritional and chemopreventive anti-cancer agents increase p27 protein expression. This apparent increase in p27 protein expression might have been due to either increased synthesis or decreased degradation, or a combination of both [1].

To address this question, the effects of various nutritional and chemopreventive anti-cancer agents on the activity of the proximal 5'-upstream region of *p27 *gene were investigated by transient transfection assay. This study provided evidence that the up-regulation of p27 protein expression is at least in part due to increased synthesis and that this increase fairly faithfully recapitulates the cancer preventive activity of nutritional and chemopreventive anti-cancer agents. Further studies were conducted to gain some insight into the molecular basis of this increase in the synthesis of p27.

## Results

### Nutritional and chemopreventive anti-cancer agents up-regulate the activity of proximal 5'-upstream region (-1797) of p27 gene in a manner specific to p27

**Figure 1:**
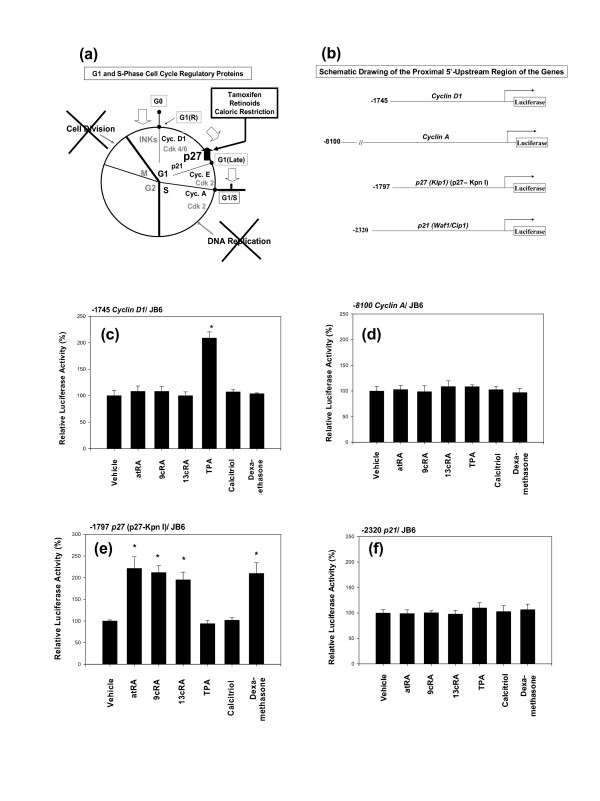
**Nutritional and chemopreventive anti-cancer agents up-regulate the activity of the proximal 5-upstream region (-1797) of *p27 *gene in a manner specific to p27**. (a) Preliminary studies suggesting – but not proving – that various nutritional and chemopreventive anti-cancer agents, including moderate caloric/dietary restriction, up-regulate p27 expression. (b) Schematic drawing of the proximal 5'-upstream region of the genes used in this experiment: (c) -1745 *cyclin D*, (d) -8100 *cyclin A*, (e) -1797 *p27 *(p27-Kpn I), and (f) -2320 *p21*. The JB6 cells, transfected with these proximal 5'-upstream region of the genes, were exposed to all-*trans*-retinoic acid (atRA) (1 μM), 9-*cis*-retinoic acid (9cRA) (1 μM), 13-*cis*-retinoic acid (13cRA) (1 μM), phorbol 12-myristate 13-acetate (TPA) (16.2 nM), 1α, 25-dihydroxyvitamin D3 (calcitriol) (240 nM), or dexamethasone (1 μM) for 24 hours. All assays were performed in triplicates and the transfection of JB6 cells with each 5'-upstream region of the genes was repeated three times.

Preliminary studies using *in vivo *model of MNU-induced rat mammary cancer and *in vitro *model of cultured cells had suggested – but not proved – that various nutritional and chemopreventive anti-cancer agents, including moderate dietary restriction, up-regulated the expression of p27 (Fig. 1a).

To prove or disprove this preliminary observation, each luciferase reporter vector containing proximal 5'-upstream region of the *cyclin D1*, *cyclin A*, *p27 *(p27-Kpn I) or *p21 *genes (Fig. 1b) was transiently transfected into promotion-sensitive (P+) JB6 mouse epidermal cells and then treated with all-*trans*-retinoic acid (atRA), 9-*cis*-retinoic acid (9cRA), 13-*cis*-retinoic acid (13cRA), phorbol 12-myristate 13-acetate (TPA), 1α, 25-dihydroxyvitamin D3 (calcitriol), or dexamethasone. Phorbol 12-myristate 13-acetate (TPA) is not a chemopreventive anti-cancer agent; rather it is a tumor promoter. But TPA was included here to demonstrate that it could stimulate the activity of the proximal 5'-upstream region (-1745) of *cyclin D1*. The TPA, three retinoic acids and dexamethasone exerted spurious effects on the backbone of the empty luciferase reporters when JB6 cells were used. Therefore, a special method, as described in the Methods and Materials section, was used to correct these effects when JB6 cells were exposed to these compounds.

The proximal 5'-upstream region (-1745) of *cyclin D1 *was activated only by TPA (Fig. 1c). Using a wild-type -963 *cyclin D1 *and a -963 *cyclin D1 *mutated at AP-1, it was confirmed that TPA activated the proximal 5'-upstream region (-1745) of *cyclin D1 *gene primarily through its TPA-response element (TRE).

The proximal 5'-upstream region of *cyclin A *and *p21 *genes were not activated by any of the compounds tested (Fig. 1d and 1f).

In contrast, the proximal 5'-upstream region (-1797) of *p27 *gene (p27-Kpn I) was activated by four nutritional and chemopreventive anti-cancer agents, namely all-*trans*-retinoic acid (atRA), 9-*cis*-retinoic acid (9cRA), 13-*cis*-retinoic acid (13cRA) and dexamethasone (Fig. 1e).

### Activation of the proximal 5'-upstream region (-1797) of p27 gene fairly faithfully recapitulates breast cancer preventive activity of various nutritional and chemopreventive anti-cancer agents

**Figure 2:**
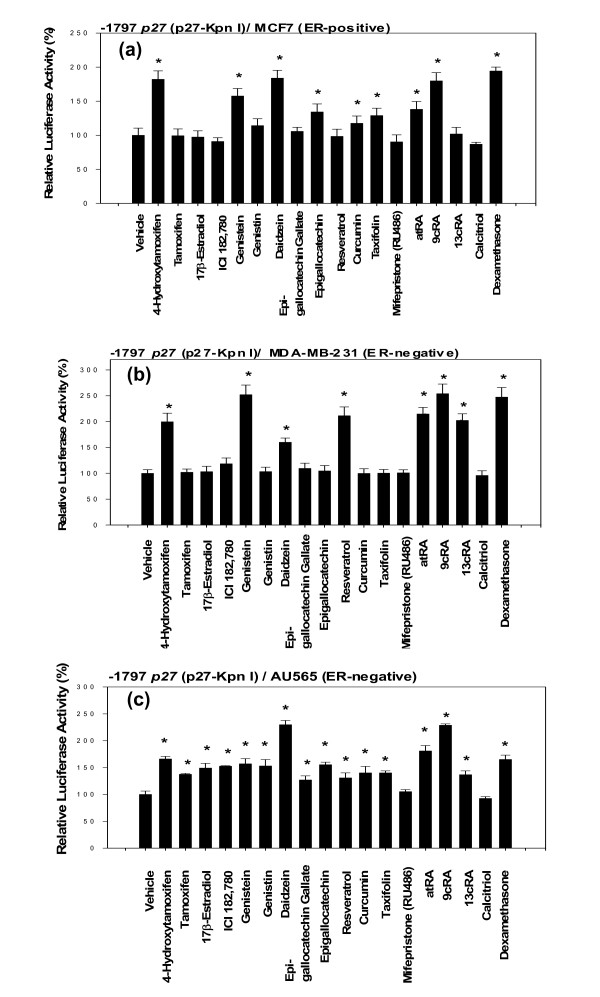
**Activation of the proximal 5'-upstream region (-1797) of *p27 *gene fairly faithfully recapitulates breast cancer preventive activity of various nutritional and chemopreventive anti-cancer agents**. (a) Estrogen-positive (ER) MCF 7, (b) ER-negative MDA-MB-231, and (c) ER-negative AU565 cells, transfected with -1797 *p27 *(p27-Kpn I), was exposed to 4-hydroxytamoxifen (258 nM), tamoxifen (269 nM), 17β-estradiol (367 nM), ICI 182 780 (165 nM), genistein (370 nM), genistin (231 nM), daidzein (393 nM), epigallocatechin-3-gallate (218 nM), epigallocatechin (326 nM), resveratrol (438 nM), curcumin (271 nM), taxifolin (329 nM), mifepristone (RU486) (233 nM), all-*trans*-retinoic acid (atRA) (1 μM), 9-*cis*-retinoic acid (9cRA) (1 μM), 13-*cis*-retinoic acid (13cRA) (1 μM), 1α, 25-dihydroxyvitamin D3 (calcitriol) (240 nM), or dexamethasone (1 μM) for 24 hours. All assays were performed in triplicates and the transfection experiments were repeated three times.

To investigate whether this specific activation of the proximal 5'-upstream region (-1797) of *p27 *gene (p27-Kpn I) recapitulates breast cancer preventive activity of various nutritional and chemopreventive anti-cancer agents, -1797 *p27 *(p27-Kpn I) was transfected into three different human breast cancer cell lines – estrogen receptor (ER)-positive MCF7 (Fig. 2a), ER-negative MDA-MB-231 (Fig. 2b), and ER-negative AU565 (Fig. 2c) – and then exposed to the following eighteen different compounds for 24 hours: 4-hydroxytamoxifen, tamoxifen, 17β-estradiol, ICI 182 780, genistein, genistin, daidzein, epigallocatechin-3-gallate, epigallocatechin, resveratrol, curcumin, taxifolin, mifepristone (RU486), all-*trans*-retinoic acid (atRA), 9-*cis*-retinoic acid (9cRA), 13-*cis*-retinoic acid (13cRA), 1α, 25-dihydroxyvitamin D3 (calcitriol), or dexamethasone. Preliminary studies indicated that none of these compounds exerted any spurious effects on the backbone of the empty luciferase reporter when human breast cancer cells were used.

4-Hydroxytamoxifen – but not tamoxifen – is the ultimate cancer preventive agents. Our results showed that 4-hydroxytamoxifen – but not tamoxifen – activated -1797 *p27 *in ER-positive MCF7 (Fig. 2a) and ER-negative MDA-MB-231 (Fig. 2b). These results indicated that (a) the activity of -1797 *p27 *recapitulated the differential breast cancer preventive efficacy of 4-hydroxytamoxifen and tamoxifen and that (b) the estrogen receptor (ER) was not involved in the activation of -1797 *p27*. In AU565 cells, both 4-hydroxytamoxifen and tamoxifen activated -1797 *p27 *(Fig. 2c), suggesting that either tamoxifen was converted to 4-hydroxytamoxifen in these cells or, as the results presented below suggest, the global rate of transcription was generally reduced in these cells, which, in turn, activated normally inactive tamoxifen by some unknown mechanisms.

Similarly, genistein – but not genistin – from soybeans is the ultimate cancer preventive agents. Our results showed that genistein – but not genistin – activated -1797 *p27 *in MCF7 (Fig. 2a) and MDA-MB-231 cells (Fig. 2b). These results again indicated that (a) the activity of -1797 *p27 *recapitulated the differential breast cancer preventive efficacy of genistein and genistin and that (b) the estrogen receptor (ER) was not involved in the activation of -1797 *p27*. In AU565 cells (Fig. 2c), however, both genistein and genistin activated -1797 *p27 *suggesting again that either genistin was converted to genistein in AU565 cells or, as the results presented below suggest, the global rate of transcription was reduced in these cells, which, in turn, activated normally inactive genistin by some unknown mechanisms.

Daidzein from soybeans activated -1797 *p27 *in all three cell lines (Fig. 2a,b,c).

Epigallocatechin – but not epigallocatechin-3-gallate – from green tea activated -1797 *p27 *in MCF7 cells (Fig. 2a), but neither epigallocatechin nor epigallocatechin-3-gallate activated -1797 *p27 *in MDA-MB-231 cells (Fig. 2b). In AU565 cells (Fig. 2c), both epigallocatechin and epigallocatechin-3-gallate activated -1797 *p27*. Resveratrol from grape skin did not activate -1797 *p27 *in MCF7 cells, but it did in MDA-MB-231 and AU565 cells. Curcumin from curry spice and taxifolin from citrus activated -1797 *p27 *in MCF7 and AU565 cells, but neither curcumin nor taxifolin activated -1797 *p27 *in MDA-MB-231 cells.

Of the three different forms of retinoic acid tested, 9-*cis*-retinoic acid (9cRA) most strongly activated -1797 *p27*, followed by all-*trans*-retinoic acid (atRA) and 13-*cis*-retinoic acid (13cRA) in all three human breast cancer cell lines. In JB6 mouse epidermal cells, these retinoic acids almost equally activated -1797 *p27 *(Fig. 1e). These results are compatible with those obtained using *in vivo *experimental animal models of breast cancer.

Dexamethasone activated -1797 *p27 *in all three human breast cancer cell lines (Fig. 2a,b,c).

Mifepristone (RU486) and 1α, 25-dihydroxyvitamin D3 (calcitriol) did not activate -1797 *p27 *in all three human breast cancer cell lines (Fig. 2a,b,c).

The estrogen receptor (ER)-negative AU565 cells were unusual in that sixteen of the eighteen compounds tested activated -1797 *p27 *(Fig. 2c); only mifepristone (RU486) and 1α, 25-dihydroxyvitamin D3 (calcitriol) did not activate it. This unusually high cancer preventive activity of nutritional and chemopreventive anti-cancer agents in AU565 cells might be due to its potentially reduced rate of transcription.

In summary, with the possible exception of AU565 cells, (a) activity of -1797 *p27 *in either estrogen receptor (ER)-positive or -negative human breast cancer cells fairly faithfully recapitulated the breast cancer preventive activity *in vivo *of the various nutritional and chemopreventive anti-cancer agents and (b) the effective doses for the activation of -1797 *p27 *by these agents were within the range that had been found effective for *in vivo *rat models of breast cancer.

### Deletion analysis indicates that various nutritional and chemopreventive anti-cancer agents activate the proximal 5'-upstream region (-1797) of p27 gene through its 5'-untranslated region (5'UTR) (-575)

**Figure 3:**
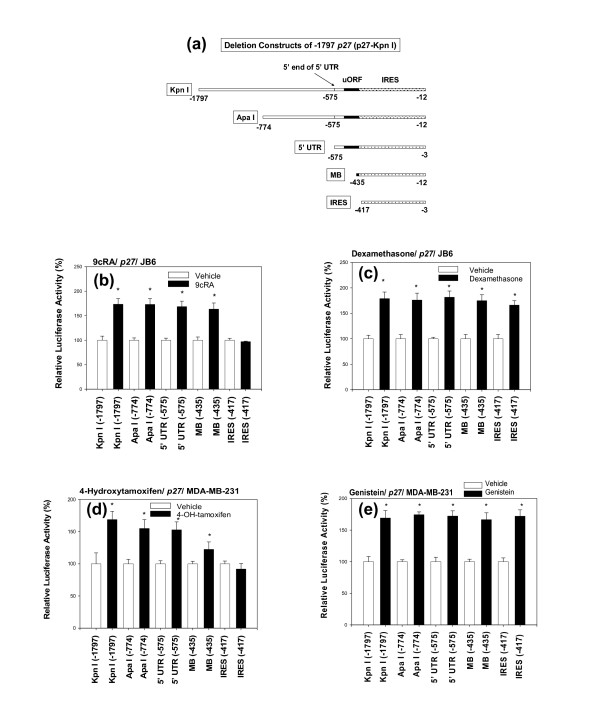
**Deletion analysis indicates that various nutritional and chemopreventive anti-cancer agents activate the proximal 5'-upstream region (-1797) of *p27 *gene through its 5'-untranslated region (5'UTR)**. (a) Deletion mutants of -1797 *p27 *(p27-Kip I) used in this experiment: -1797 *p27 *(p27-Kpn I), -774 *p27 *(p27-Apa I), -575 *p27 *(p27-5'UTR), -435 *p27 *(p27-MB), and -417 *p27 *(p27-IRES). The (P+) JB6 mouse epidermal cells and estrogen receptor (ER)-negative MDA-MB-231 cells were transfected with these deletion mutants and then exposed to (b) 9-*cis*-retinoic acid (9cRA) (1 μM), (c) dexamethasone (1 μM), (d) 4-hydroxytamoxifen (258 nM), or (e) genistein (370 nM) for 24 hours. All assays were performed in triplicates and the transfection experiments were repeated three times.

To determine the core element for the activation of the proximal 5'-upstream region (-1797) of *p27 *gene, (P+) JB6 mouse epidermal cells or estrogen receptor (ER)-negative MDA-MB-231 human breast cancer cells were transfected with the following deletion mutants of -1797 *p27 *(Fig. 3a) and then treated with various nutritional and chemopreventive anti-cancer agents: -1797 *p27 *(p27-Kpn I) [4], -774 *p27 *(p27-Apa I) [4], -575 *p27 *(p27-5'UTR) [5,6], -435 *p27 *(p27-MB) [4], and -417 *p27 *(p27-IRES) [5,6]. Preliminary studies indicated that TPA, three retinoic acids and dexamethasone exerted spurious effects on the backbone of the empty luciferase reporters when JB6 cells were used. Therefore, special method, as described in the Methods and Materials section, was used to normalize these effects in these cases. When human breast cancer cells were used, none of the agents tested exerted any spurious effects on the backbone of the empty luciferase reporters.

The results of deletion analysis suggested that, in all cases without exception (Fig. 3b to 3e), including those not shown here, the various nutritional and chemopreventive anti-cancer agents activated proximal 5'-upstream region (-1797) of *p27 *gene at least through -575 *p27 *(5'-untranslated region (5'UTR) of *p27 *gene). When the regions shorter than -575 *p27 *(p27-5'UTR) were tested, the activities tended to either stay constant or be reduced; the activities never increased above that of -575 *p27 *(p27-5'UTR).

### The -575 p27 (5'-untranslated region (5'UTR) of p27 gene) is unlikely to contain any cryptic transcription factor binding sites

**Figure 4:**
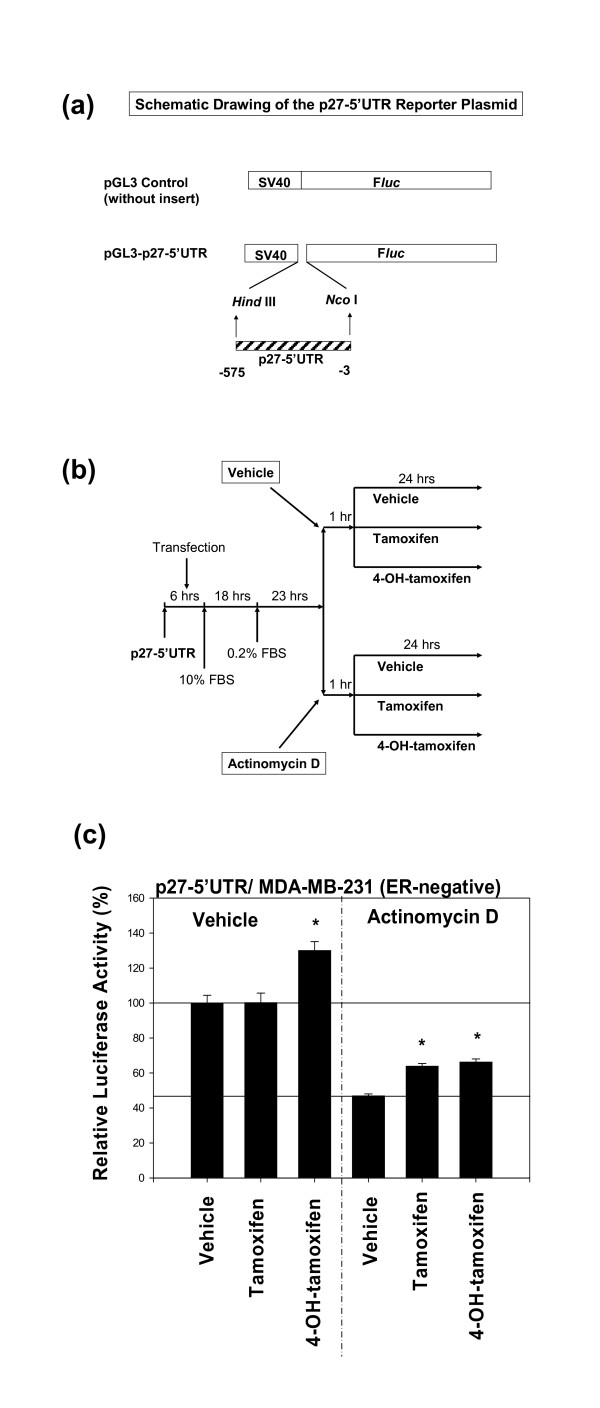
**It is unlikely that -575 p27 (5'-untranslated region (5'UTR) of *p27 *gene) contains any cryptic transcription factor binding sites**. (a) Schematic drawing (adapted from references [5,6]) of the pGL3-control-p27-5'UTR-luciferase reporter plasmid. (b) Summary of the experimental protocol. (c) The ER-negative MDA-MB 231 cells were transfected with -575 *p27 *(p27-5'UTR) and then either vehicle or actinomycin D (0.5 μg/ml) [29] was added to the medium. One hour after the addition of these compounds, the cells were exposed to vehicle, tamoxifen (1.076 μM) or 4-hydroxytamoxifen (1.032 μM) for 24 hours. All assays were performed in triplicates and the transfection experiments were repeated three times.

To investigate if -575 *p27 *(p27-5'UTR) contains any cryptic transcription factor binding sites, the activity of the region was stimulated with 4-hydroxytamoxifen in the presence of an antibiotic actinomycin D. Figure 4a shows the schematic drawing of the pGL3 control-p27-5'UTR-luciferase reporter plasmid. This plasmid – pGL3 control – contains SV40 promoter in its backbone. Preliminary tests using pGL3 control without p27-5'UTR insert had demonstrated that 4-hydroxytamoxifen, tamoxifen, or vehicle (DMSO) did not exert any spurious effects on the SV40 promoter when human breast cancer cells were used. The outline of the protocol for actinomycin D experiment is shown in Figure 4b. Actinomycin D was added one hour before the addition of 4-hydroxytamoxifen, tamoxifen, or vehicle (DMSO) and then the cells were kept exposed to both compounds for another 24 hours.

The results (Fig. 4c) indicated that in the absence of actinomycin D, only 4-hydroxytamoxifen significantly up-regulated the activity of -575 *p27 *(p27-5'UTR) above that of vehicle (DMSO) in MDA-MB-231 cells; tamoxifen did not up-regulate it. The addition of actinomycin D in the presence of vehicle (DMSO) alone decreased the baseline activity of -575 *p27 *(p27-5'UTR) by about 53% relative to the activity observed in the absence of actinomycin D. Despite this decrease in the baseline activity in the presence of actinomycin D, 4-hydroxytamoxifen still significantly up-regulated the activity of -575 *p27 *(p27-5'UTR) above that of vehicle (DMSO). These findings suggested that transcriptional mechanisms were not involved in the up-regulation of the activity of -575 *p27 *(p27-5'UTR) by 4-hydroxytamoxifen, precluding the involvement of any cryptic transcription binding sites in this region. What was more surprising was the finding that tamoxifen, which had previously been inactive in the absence of actinomycin D, significantly up-regulated the activity of -575 *p27 *(p27-5'UTR) in the presence of actinomycin D, suggesting that the overall level of global transcriptional rate might somehow modulate the effects of tamoxifen on the activity of -575 *p27 *(p27-5'UTR) in MDA-MB-231 cells.

### Inhibition of certain receptor protein tyrosine kinases (RPTKs) up-regulates the activity of -575 p27 (5'-untranslated region (5'UTR) of p27 gene)

The results presented above suggested that the estrogen receptor was not involved in the activation of -1797 *p27 *(p27-Kpn I) by 4-hydroxytamoxifen, genistein, daidzein and probably other nutritional and chemopreventive anti-cancer agents. Tamoxifen and genistein have been known to exhibit anti-estrogenic activity, but in addition, they have been reported to inhibit receptor protein tyrosine kinase (RPTK) activity at a slightly higher concentrations [9,10]. Epigallocatechin-3-gallate has also been reported to block activation of RPTKs such as epidermal growth factor receptor (EGFR) and HER-2/neu receptor, which are generally overexpressed or constitutively active in many human malignancies [11,12].

Although multiple RPTKs are known to be expressed in human breast cancer cells, synthetic inhibitors of four RPTKs – epidermal growth factor receptor (EGFR), HER/ErbB, platelet-derived growth factor receptor (PDGFR) and insulin receptors (IR and IGF-1R) – were used to investigate whether they up-regulate the activity of -575 *p27 *(p27-5'UTR). Preliminary studies had demonstrated again that none of them exerted any spurious effects on the backbone of the empty luciferase reporter plasmids in all four types of cells used in this experiment.

**Figure 5:**
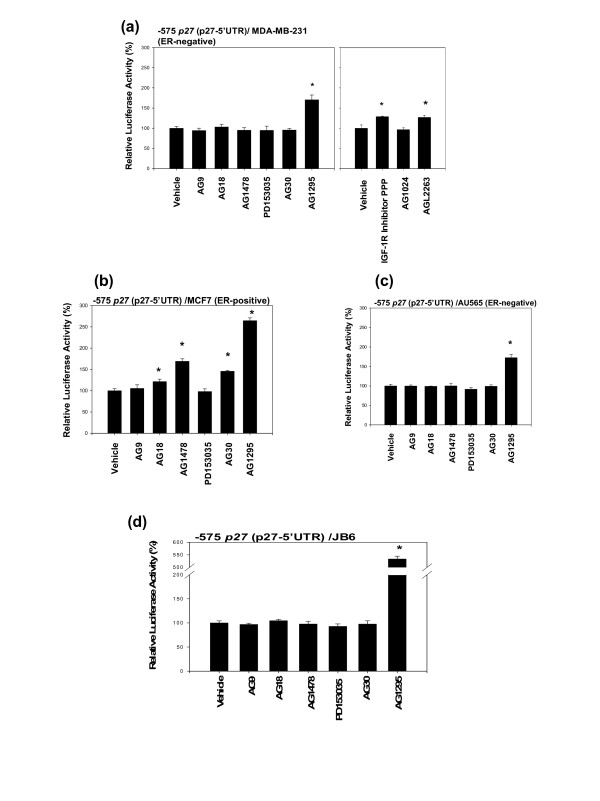
**Inhibition of certain receptor protein tyrosine kinases (RPTKs) up-regulates the activity of -575 *p27 *(5'-untranslated region (5'UTR) of p27 gene)**. (a) MDA-MB-231, (b) MCF7, (c) AU565, and (d) JB6 cells were transfected with -575 *p27 *(p27-5'UTR) and then exposed to inhibitors of RPTKs for 24 hours. The following inhibitors were used: AG9 (2.71 μM), AG18 (2.69 μM), AG1478 (1.58 μM), and PD153035 (1.39 μM) to inhibit epidermal growth factor receptor (EGFR); AG30 (2.44 μM) to inhibit c-ErbB; AG1295 (2.13 μM) to inhibit platelet-derived growth factor receptor (PDGFR); IGF-1R inhibitor PPP (1.21 μM), AG1024 (1.64 μM), and AGL2263 (1.55 μM) to inhibit insulin receptors (IR) and type 1 insulin-like growth factor receptors (IGF-1R). All assays were performed in triplicates and the transfection experiments were repeated three times.

The following four synthetic inhibitors were used to inhibit EGFR: AG9 (inactive negative control for inhibition of EGFR), AG18 (broad spectrum protein tyrosine kinase inhibitor, inhibits EGFR autophosphorylation), AG1478 (specific inhibitor of EGFR), and PD153035 (specific inhibitor of tyrosine kinase activity of EGFR). Of the four inhibitors, two of them – AG18 and AG1478 – up-regulated the activity of -575 *p27 *(p27-5'UTR) in MCF7 cells (Fig. 5b). However, none of them up-regulated the activity of -575 *p27 *(p27-5'UTR) in MDA-MB-231 (Fig. 5a), AU565 (Fig. 5c), and JB6 cells (Fig. 5d).

AG30, a specific inhibitor of c-ErbB, did not up-regulate the activity of -575 *p27 *(p27-5'UTR) in any of the cells tested (Fig. 5a to 5d).

In contrast, AG1295, a specific inhibitor of PDGFR, up-regulated the activity of -575 *p27 *(p27-5'UTR) in all four types of cells (Fig. 5a to 5d).

Three inhibitors of insulin receptors were investigated using MDA-MB-231 cells (Fig. 5a). Of the three inhibitors, two of them – IGF-IR inhibitor PPP and AGL2263 (IR and IGF-1R inhibitor) – up-regulated the activity of -575 *p27 *(p27-5'UTR) in MDA-MB-231 cells, but AG1024 (IGF-1 inhibitor) failed to up-regulate it.

Taken together, these results suggested that inhibition of certain RPTKs on the cell surface could up-regulate the activity of -575 *p27 *(p27-5'UTR).

### Inhibition of certain mitogen-activated protein kinases (MAPKs) up-regulates the activity of -575 p27 (5'-untranslated region (5'UTR) of p27 gene)

When the cell-surface RPTKs are inhibited, the inhibitory signal could be transmitted to the interior of the cells in two ways. The inhibition of mitogen-activated protein kinases (MAPKs) is one of them. Therefore, inhibitors of MAPKs were used to investigate whether any one or more of them could up-regulate the activity of -575 *p27 *(p27-5'UTR). Again, none of them exerted any spurious effects on the backbone of the empty luciferase reporter plasmids in all four types of cells used in this experiment.

**Figure 6:**
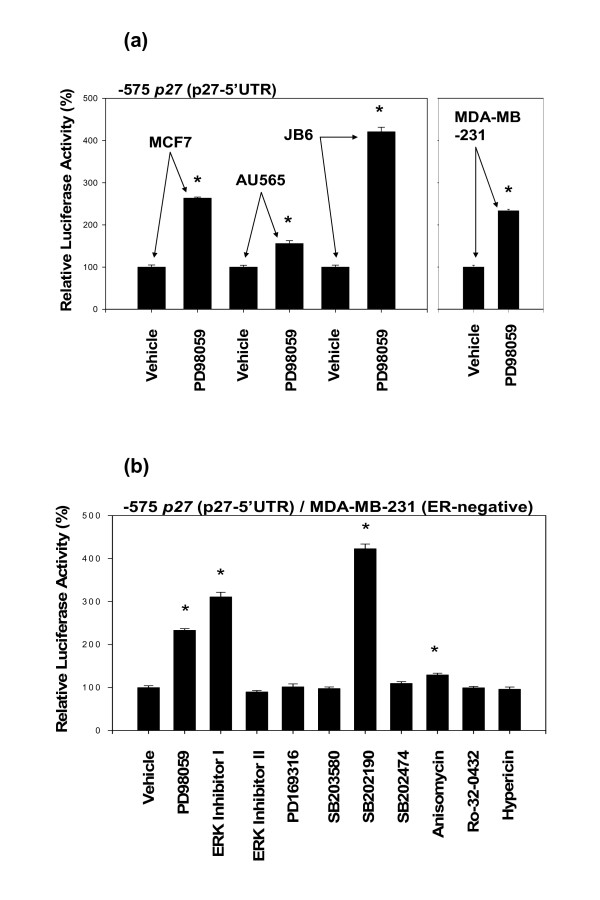
**Inhibition of certain mitogen-activated protein kinases (MAPKs) up-regulates the activity of -575 *p27 *(5'-untranslated region (5'UTR) of *p27 *gene)**. All four types of cells were transfected with -575 *p27 *(p27-5'UTR) and then exposed to the following inhibitors: PD98059 (1.87 μM) to inhibit MEK; ERK activation inhibitor peptide I (0.80 μM) and ERK activation inhibitor peptide II (1.42 μM) to inhibit ERK; PD169316 (1.39 μM), SB203580 (1.33 μM), SB202190 (1.51 μM), and SB202474 (1.79 μM) to inhibit p38MAPK; anisomycin (1.89 μM) to activate MAPKs and stress-activated proteins kinases (SAPKs); Ro-32-0432 (1.02 μM) to inhibit protein kinase C (PKC); and hypericin (0.99 μM) to inhibit PKC/ERK. All assays were performed in triplicates and the transfection experiments were repeated three times.

PD98059, an inhibitor of MEK, up-regulated the activity of -575 *p27 *(p27-5'UTR) in all four types of cells tested (Fig. 6a). The effects of other inhibitors of MAPKs were investigated using MDA-MB-231 cells (Fig. 6b). Of the two inhibitors of ERK tested, ERK activation inhibitor peptide I up-regulated the activity of -575 *p27 *(p27-5'UTR) in MDA-MB-231 cells, but ERK activation inhibitor peptide II did not up-regulate it. And of the four p38MAPK inhibitors, only SB202190 strongly up-regulated the activity of -575 *p27 *(p27-5'UTR); the other three inhibitors – PD169316, SB203580 and SB202474, an inactive negative control for p38MAPK inhibitors – failed to up-regulate it. Anisomycin, which activates MAPKs and stress-activated protein kinases (SAPKs), also slightly up-regulated the activity of -575 *p27 *(p27-5'UTR), and the other two inhibitors – Ro-32-0432 (protein kinase C (PKC) inhibitor) and hypericin (PKC/ERK inhibitor) – did not have any significant effects on the activity of -575 *p27 *(p27-5'UTR).

### Inhibition of phosphoinositide 3-kinase/Akt/mammalian target of rapamycin (PI3K/Akt/mTOR) also up-regulates the activity of -575 p27 (5'-untranslated region (5'UTR) of p27 gene)

The signal that the cell-surface RPTKs are inhibited is also transmitted to the interior of the cells by phosphoinositide 3-kinase/Akt/mammalian target of rapamycin (PI3K/Akt/mTOR) pathway. Therefore, inhibitors of these three protein kinases were used next to investigate whether they also up-regulate the activity of -575 p27 (p27-5'UTR) in MDA-MB-231 cells. Again, preliminary experiments had been conducted to verify that none of them had exerted any spurious effects on the backbone of the empty luciferase reporter plasmids in MDA-MB-231 cells.

**Figure 7:**
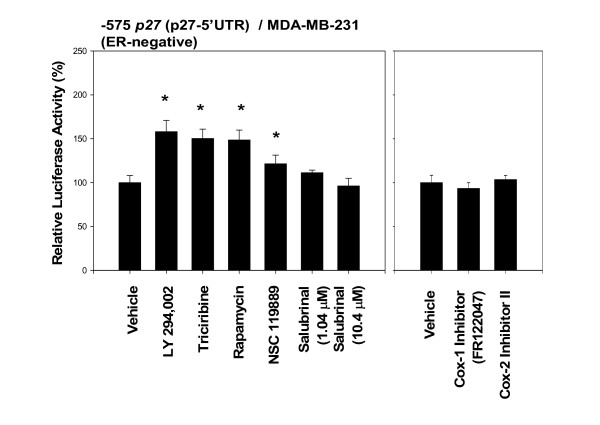
**Inhibition of phosphoinositide 3-kinase/Akt/mammalian target of rapamycin (PI3K/Akt/mTOR) also up-regulates the activity of -575 *p27 *(5'-untranslated region (5'UTR) of *p27 *gene). Furthermore, inhibition of the global methylation of 5'-m^7^G-cap of other mRNAs also up-regulates the activity of -575 *p27 *(5'-untranslated region (5'UTR) of p27 gene)**. The estrogen receptor (ER)-negative MDA-MB-231 cells, transfected with -575 *p27 *(p27-5'UTR), were treated with LY 294002 (3.25 μM) to inhibit PI3K; triciribine (3.12 μM) to inhibit Akt/PKB, rapamycin (1.09 μM) to inhibit mTOR; COX-1 inhibitor FR122047 (1.09 μM) and COX-2 inhibitor II (1.43 μM) to inhibit cyclooxygenases; NSC 119889 (7.70 μM) to competitively inhibit S-adenosylmethionine; or salubrinal (1.04 and 10.4 μM) to inhibit phosphatase of eukaryotic translation initiation factor 2α (eIF2α) for 24 hours. All assays were performed in triplicates and the transfection experiments were repeated three times.

The results indicated that LY294,002 (an inhibitor of PI3K) [5], triciribine (Akt inhibitor) and rapamycin (mTOR inhibitor) all three of them up-regulated the activity of -575 *p27 *(p27-5'UTR) (Fig. 7).

Also shown in Figure 7 are the effects of two cyclooxygenase inhibitors – COX-1 inhibitor FR122047 and COX-2 inhibitor II – both of which failed to up-regulate the activity of -575 *p27 *(p27-5'UTR) in MDA-MB-231 cells.

### Inhibition of the global methylation of 5'-m^7^G-cap of mRNAs also up-regulates the activity of -575 p27 (5'-untranslated region (5'UTR) of p27 gene)

There are two ways to suppress the global cap-dependent translation initiation of 5'-m^7^G-capped mRNAs, thereby potentially up-regulating the activity of -575 *p27 *(p27-5'UTR) by a set of mechanisms called cap-independent translation initiation. One way is to inhibit the methylation of 5'-m^7^G cap of mRNAs by S-(5'-adenosyl)-L-methionine (AdoMet or SAM). Another way is to stimulate the inhibitory effect of endoplasmic reticulum stress by increasing the phosphorylation of the eukaryotic translation initiation factor 2α (eIF2α). The results (Fig. 7) indicated that NSC 119889, a cell-permeable, competitive inhibitor of AdoMet (SAM) and which also acts as a global inhibitor of cap-dependent translation initiation, up-regulated the activity of -575 *p27 *(p27-5'UTR) in estrogen receptor (ER)-negative MDA-MB-231 cells. The salubrinal, a cell-permeable thiourea compound, that acts as a selective inhibitor of eukaryotic translation initiation factor 2α (eIF2α) dephosphorylation by phosphatase complex, failed to up-regulate the activity of -575 *p27 *(p27-5'UTR) in MDA-MB-231 cells (Fig. 7). Again, both inhibitors had not exerted any spurious effects on the backbone of the empty luciferase reporter plasmids in MDA-MB-231.

### Modulation of the phosphorylation of 5'-AMP-activated protein kinase (AMPK; a metabolic energy sensor or cellular fuel gauge) either up-regulates or down-regulates the activity of -575 p27 (p27-5'UTR)

5'-AMP-activated protein kinase, known as AMPK, acts as a metabolic energy sensor or cellular fuel gauge playing a key role in the regulation of energy metabolism [13]. A number of physiological and pathophysiological stimuli, including caloric restriction, lead to an increase in the AMP: ATP ratio within the cell, which activates AMPK by phosphorylating it at Thr172 in the activation loop. When activated, AMPK modulates tuberous sclerosis complex (TSC), down-regulates phosphorylation of mammalian target of rapamycin (mTOR), eukaryotic translation initiation 4E binding protein 1 (4EBP1) and p70 S6 kinase (S6K) [13,14,30]. Thus, caloric restriction – by way of AMPK, TSC, mTOR, and 4EBP1 – might potentially up-regulate the activity of -575 *p27 *(p27-5'UTR).

**Figure 8:**
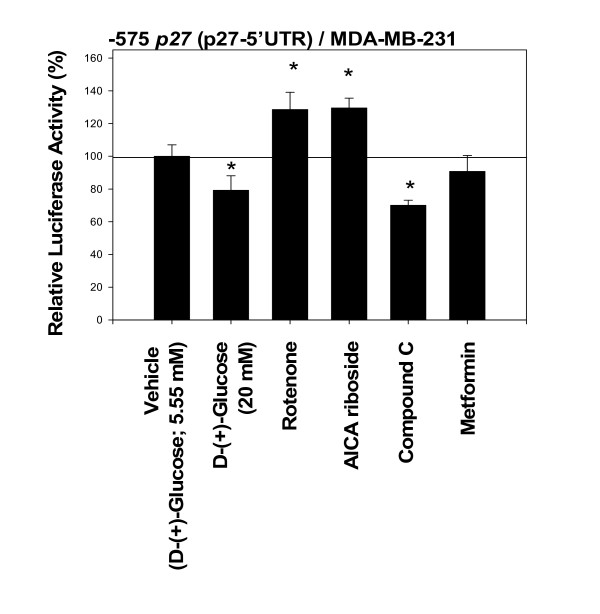
**Modulation of 5'-AMP-activated protein kinase (AMPK; a metabolic energy sensor or cellular fuel gauge) pathway either up-regulates or down-regulates the activity of -575 *p27 *(p27-5'UTR)**. The estrogen receptor (ER)-negative MDA-MB-231 cells were transfected with -575 *p27 *(p27-5'UTR) and then exposed to D-(+)-glucose (20 mM), rotenone (20 μM), or AICA riboside (50 μM), compound C (1.25 μM) or metformin (3.02 μM) for 24 hours. The basal culture medium (DMEM) contained 1 g/L (or 5.55 mM) rather than the usual 4.5 g/L (or 25 mM) of D-(+)-glucose. All assays were performed in triplicates and the transfection experiments were repeated three times.

**Figure 9:**
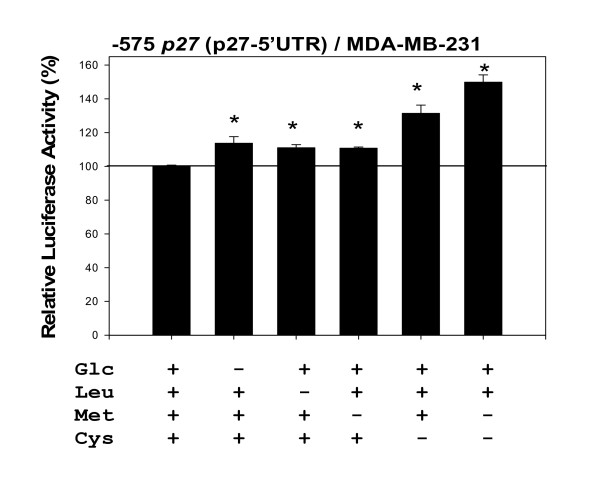
**Amino acid and glucose deficiencies up-regulate the activity of -575 *p27 *(p27-5'UTR)**. The MDA-MB-231 cells were transfected with -575 *p27 *(p27-5'UTR) and then maintained in 0.2% FBS DMEM for 24 hours. At this point in time, the medium was changed to a DMEM Labeling Kit purchased from Chemicon International (Temecula, CA, USA). This was a deficient basal DMEM supplemented with appropriate components to prepare complete DMEM and DMEM without D-(+)-glucose, L-leucine, L-methionine, L-cysteine, or combination of L-methionine and L-cysteine. Complete DMEM contained 4.5 g/L D-(+)-glucose, 105 mg/L L-leucine, 30 mg/L L-methionine, and 48 mg/L L-cysteine. The cells were maintained in these complete and deficient DMEMs for 24 hours. All assays were performed in triplicates and the transfection experiments were repeated three times.

The Figure 8 shows the effects of various modulators of AMPK phosphorylation on the activity of -575 *p27 *(p27-5'UTR) in MDA-MB-231 cells. Again, none of the compounds tested had exerted any spurious effects on the backbone of the empty luciferase reporter plasmids in MDA-MB-231 cells. The results indicated that two compounds – rotenone (an inhibitor of mitochondrial electron transport) and AICA riboside (an analogue of 5'-AMP) – that are known to increase phosphorylation of AMPK up-regulated the activity of -575 *p27 *(p27-5'UTR) (also see a recent report [35]). The results shown in Figure 9 also demonstrated that the removal of D-(+)-glucose from the cell culture medium up-regulated the activity of -575 *p27 *(p27-5'UTR). In contrast, the compounds that are known to decrease phosphorylation of AMPK – excess D-(+)-glucose and compound C – down-regulated the activity of -575 *p27 *(p27-5'UTR).

Metformin is a widely used drug for treatment of type 2 diabetes with no defined cellular mechanism of action. It has been suggested recently that metformin could increase phosphorylation of AMPK [15,38,39]. However, the results, as shown in Figure 8, indicated that metformin did not up-regulate the activity of -575 *p27 *(p27-5'UTR) in MDA-MB-231 cells.

### Amino acid deficiency up-regulates the activity of -575 p27 (p27-5'UTR)

In addition to caloric restriction and growth factor signals, tuberous sclerosis complex (TSC) could transmit amino acid deficiency signals to regulate mTOR activity [13]. We already demonstrated that rapamycin, an inhibitor of mTOR phosphorylation, up-regulated the activity of -575 *p27 *(p27-5'UTR) in MDA-MB-231 cells (see Fig. 7). Therefore, the effects of amino acid deficiency on the activity of -575 *p27 *(p27-5'UTR) were investigated using MDA-MB-231 cells. Again, none of the amino acid deficiencies tested had exerted any spurious effects on the backbone of the empty luciferase reporter plasmids in MDA-MB-231 cells. As shown in Figure 9, removal of L-leucine, L-methionine, L-cysteine, or combination of L-methionine and L-cysteine, all up-regulated the activity of -575 *p27 *(p27-5'UTR) in MDA-MB-231 cells. The findings of L-methionine deficiency are interesting because L-methionine deficiency could up-regulate the activity of -575 *p27 *(p27-5'UTR) in two ways: one is to decrease methylation of 5'-m^7^G cap of mRNAs and another is to decrease phosphorylation of mTOR by TSC.

## Discussion

The results of the study presented above indicated that various nutritional and chemopreventive anti-cancer agents up-regulate the expression of p27 in mouse epidermal (JB6) and human breast cancer (MCF7, MDA-MB-321, and AU565) cells. Up-regulation of the expression of p27 – measured by the activity of the proximal 5'-upstream region (-1797) of *p27 *gene (p27-Kpn I) – appears to be specific to p27 because expression of cyclin D1, E, and A, and p21Cip1/Waf1, another cyclin-dependent kinase inhibitor of G1-to-S phase transition, was not affected by these agents. Furthermore, up-regulation of the activity of the proximal 5'-upsteam region (-1797) of *p27 *gene (p27-Kpn I) fairly faithfully recapitulated the breast cancer preventive activity of various nutritional and chemopreventive anti-cancer agents.

Deletion analysis of the proximal 5'-upstream region (-1797) of *p27 *gene (p27-Kpn I) indicated that the activities were maintained fairly constant – neither increased nor decreased significantly – when -1797 *p27 *(p27-Kpn I), -774 *p27 *(p27-Apa I) and -575 *p27 *(p27-5'UTR) were used. When the fragments shorter than -575 *p27 *(p27-5'UTR) were used, the activities were either maintained constant or decreased. Although transcription of the *p27 *gene to mRNA is likely to begin at the 5'-upstream end (-575) of the 5'-untranslated region (5'UTR) of the *p27 *gene, it has been claimed that there could be some cryptic transcription factor binding sites within the 5'-untranslated region (5'UTR) of the *p27 *gene (p27-5'UTR). The general issue here is whether the up-regulation of the activity of -575 *p27 *(p27-5'UTR) is due to transcriptional or translational mechanism. In the earlier literature of p27 on this issue, there used to be a general consensus that changes in p27 protein levels do not correlate with changes in the transcriptional rate of *p27 *gene. Rather, p27 expression appears to be regulated either by changes in the rate of proteasome-mediated degradation and/or the rate at which p27 mRNA is translated [16-18]. To gain some insight into this issue, the activity of -575 *p27 *(p27-5'UTR) was measured in the presence of both 4-hydroxytamoxifen and the antibiotic actinomycin D, an inhibitor of transcription. The results indicated that actinomycin D did not attenuate the up-regulation of the activity of 5'-untranslated region (5'UTR) of *p27 *gene. This finding could preclude the presence of cryptic transcription factor binding site(s) in this region and favor the translational, rather than transcriptional, theory of up-regulation of *p27 *gene. The actinomycin D experiment provided another unexpected finding. Tamoxifen, which, unlike 4-hydroxytamoxifen, had not up-regulated the activity of -575 *p27 *(p27-5'UTR) in the absence of actinomycin D, up-regulated it in the presence of actinomycin D. This finding raised the interesting question of whether the decreased level of global transcription rate could also be one of the factors that contribute to the up-regulation of the activity of -575 *p27 *(p27-5'UTR). In fact, in one of the human breast cancer cells (AU565), both 4-hydroxytamoxifen and tamoxifen were observed to up-regulate the activity of -1797 *p27 *(p27-Kpn I), suggesting that the global transcriptional rate could be lower in these cells compared to other human breast cancer cells (MDA-MB-231 and MCF7).

If we assume that the activity of 5'-untranslated region (5'UTR) of the *p27 *gene is up-regulated by a translational mechanism, what could be the molecular basis of this mechanism? Nearly all nucleus-encoded eukaryotic proteins are translated from their respective mRNAs by a mechanism involving recognition of the 5'-m^7^G cap of the mRNAs by eukaryotic translation initiation factor 4E (eIF4E). In quiescent cells eIF4E activity is repressed, leading to a global decline in translational rate. In contrast to the translation of global mRNAs, translation of p27 mRNA is highest during quiescence, suggesting that it escapes the general repression of translational initiation. It was shown that the 5'-untranslated region (5'UTR) of the p27 mRNA mediates cap-independent translation initiation [19] and, within the 5'UTR of p27 mRNA, a U-rich element (polypyrimidine site in the putative internal ribosome entry site (IRES)) [5,20] and upstream open reading frame (uORF) [6] were reported to be necessary for the cap-independent translation initiation of p27 mRNA.

**Figure 10:**
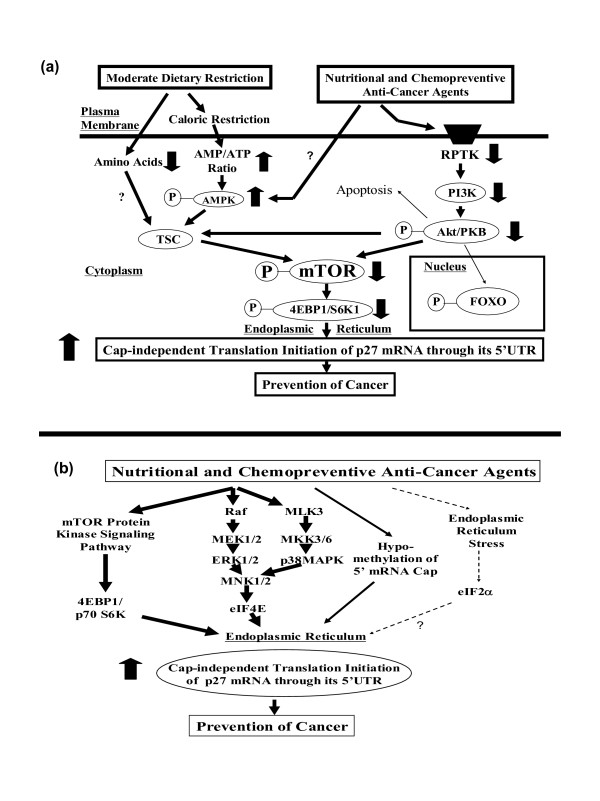
**Schematic drawings of the hypothetical signaling pathways that could lead to activation of the unusually long 5'-untranslated region (-575) of p27 mRNA**. The two signaling pathways shown in (10a) are (i) 5'-AMP-activated protein kinase (metabolic energy sensor or cellular fuel gauge)/tuberous sclerosis complex/mammalian target of rapamycin (AMPK/TSC/mTOR) and (ii) receptor protein tyrosine kinases/phosphoinositide 3-kinase/Akt/mammalian target of rapamycin (RPTKs/PI3K/Akt/mTOR). The four signaling pathways shown in (10b) are (i) the summary of the two signaling pathways indicated above, (ii) receptor protein tyrosine kinases/MAPKs (RPTKs/MAPKs), and (iii) global hypomethylation of the 5'-m^7^G cap of mRNAs.

Assuming that the activity of 5'-untranslated region (5'UTR) of *p27 *gene is up-regulated by translational rather than transcriptional mechanisms, further experiments were conducted, using -575 *p27 *(p27-5'UTR) and various putative inhibitors of signaling pathways, to gain some insight into the basic mechanisms of how various nutritional and chemopreventive anti-cancer agents could transmit their signals to activate translation initiation of p27 mRNA. The results of these experiments suggested that there are at least four signaling pathways – two of them shown in Figure 10a and all four of them in Figure 10b – that could potentially transmit the signals to activate translation initiation of p27 mRNA.

### Caloric restriction and amino acid deficiencies are likely to transmit signals to activate translation initiation of p27 mRNA via 5'-AMP-activated protein kinase (AMPK, metabolic energy sensor, or cellular fuel gauge), tuberous sclerosis complex (TSC), mammalian target of rapamycin (mTOR), and eukaryotic translation initiation factor 4E binding protein 1 (4EBP1) and p70 S6 kinase (AMPK/TSC/mTOR/4EBP1 and p70 S6K)

Caloric restriction could up-regulate translation initiation of p27 mRNA through its 5'-untranslated region (p27-5'UTR) by sending the signal to endoplasmic reticulum via AMPK, TSC, mTOR, and 4EBP/S6K. Amino acid deficiencies could also send the signals to endoplasmic reticulum via TSC, mTOR, and 4EBP/S6K. Deficiency of L-methionine is an interesting case because it could also up-regulate the cap-independent translation initiation of p27 mRNA by down-regulating global methylation of the 5'-m^7^G-cap of other mRNAs.

Caloric restriction has long been known to activate AMPK. The AMPK system is controlled by the balance between ATP consumption (e.g., by biosynthesis, cell growth, or muscle contraction) and ATP production via catabolism [21,22]. If the rate of ATP consumption exceeds its rate of production, such as during caloric restriction, ADP will tend to rise and be converted to AMP by the enzyme adenylate kinase. The rise in level of the activating ligand AMP, coupled with the fall in level of the inhibitory nucleotide ATP, activates AMPK, which then switches off ATP-consuming processes and switches on catabolism in an attempt to redress the balance.

AMPK, when activated, phosphorylates tuberous sclerosis complex 2 (TSC2), thereby inhibiting mTOR activation [13]. In addition to changes in the intracellular AMP/ATP ratio, the TSC1 (hamartin)-TSC2 (tuberin) complexes might mediate amino acid signals to regulate mTOR activity [13].

In mammalian cells, mTOR generally regulates translation. Eukaryotic translation initiation factor 4E (eIF4E) binding protein 1 (4EBP1) [30] and ribosomal p70 S6 kinase (S6K), the most extensively studied substrates of mTOR, are key regulators of protein translation [13].

4EBP1 acts as a translational repressor by binding and inhibiting the eIF4E, which recognizes the 5'-end m^7^G cap of eukaryotic mRNAs. Phosphorylation of 4EBP1 by mTOR results in a dissociation of 4EBP1 from eIF4E, thereby relieving the inhibition of 4EBP1 on eIF4E-dependent (cap-dependent) translation initiation [30].

The inhibition of mTOR, therefore, results in decreased global cap-dependent translation initiation of 5'-m^7^G-capped mRNA, but it could also increase cap-independent translation initiation of p27 mRNA through its 5'UTR.

### Nutritional and chemopreventive anti-cancer agents could also up-regulate translation of p27 by transmitting inhibitory signals to receptor protein kinases (RPTKs) followed by (a) phosphoinositide 3-kinase/Akt/mammalian target of rapamycin (PI3K/Akt/mTOR) signaling pathway, (b) MAPK/MNK signaling pathway or (c) a combination of both

Following growth factor activation of RPTKs, phosphoinositide 3-kinase (PI3K) is recruited to the receptor and activated resulting in the production of phosphatidylinositol-3,4,5-trisphosphate (PIP_3_) [23]. This recruits Akt/PKB to the membrane where it is phosphorylated by phosphoinositide-dependent kinase 1 (PDK1). Akt/PKB is then released from the membrane and translocate to other subcellular compartments.

Akt/PKB is involved in mTOR activation by phosphorylating mTOR at Ser2448 [13,24]. It is not yet settled whether Akt/PKB activates mTOR directly or indirectly, but recent biochemical studies indicated that Akt/PKB directly phosphorylates TSC2 and inhibits its function [13]. TSC2 inactivation by Akt/PKB may also inhibit mTOR indirectly through inhibition of the small GTPase, Rheb [13].

The inhibition of RPTKs, therefore, leads to reduction of the phosphorylation of mTOR and 4EBP/S6K, thereby attenuating the global cap-dependent translation initiation of 5'-m^7^G-capped mRNAs, but at the same time activating the cap-independent translation initiation of p27 mRNA through its 5'UTR.

The inhibitory signals originating from RPTKs could also be transmitted to MAPK/MNK signaling pathway. It is known that the activity of eIF4E is regulated not only by interaction with 4EBP1 but also phosphorylation by mitogen-activated protein (MAP) kinase-interacting kinase (MNK) on Ser209. The phosphorylation of eIF4E via MNK is mediated by the activation of either the ERK or p38 pathway [25]. The results presented above indicated that the inhibition of the MEK (an upstream MAPK of ERK), ERK or p38MAPK could also decrease the phosphorylation of eIF4E, thereby reducing the global cap-dependent translation initiation of 5'-m^7^G-capped mRNAs, but at the same time activating the cap-independent translation initiation of p27 mRNA through its 5'UTR.

### Global hypomethylation of the 5'-m^7^G cap of other mRNAs could also transmit signals to activate cap-independent translation initiation of p27 mRNA through its 5'UTR

Nearly all mRNAs are post-transcriptionally modified at their 5' and 3' ends, by capping and polyadenylation, respectively [26-28]. The m^7^G-capping at their 5' end protects the nascent pre-mRNAs against degradation and failure to cap or loss of cap leads to rapid breakdown of the mRNAs. The mRNA cap (guanine-N7) methyltransferase catalyzes methyl transfer from S-adenosylmethionine (AdoMet or SAM) to GpppRNA to form m^7^GpppRNA.

The results presented above indicated that the NSC 119889, a cell-permeable, competitive inhibitor of AdoMet (SAM), inhibits global cap-dependent translation initiation of 5'-m^7^G-capped mRNAs, but it could also increase cap-independent translation initiation of p27 mRNA through its 5'UTR. This finding suggests that the epigenetic methylation hypothesis of cancer should be based not only on DNA methylation but also on mRNA methylation.

Phosphorylation of the α subunit of eukaryotic translation initiation factor 2 (eIF2α) is a well-documented mechanism of down-regulating protein synthesis under a variety of stress conditions, but at the same time it could also up-regulate the cap-independent translation initiation of p27 mRNA through its 5'UTR. However, contrary to this expectation, the results presented above indicated that salubrinal, a cell-permeable thiourea compound, that acts as a selective inhibitor of translation initiation factor 2α (eIF2α) dephosphorylation by phosphatase complex, failed to up-regulate the activity of 5'-untranslated region of *p27 *gene.

### Cross-talk between 5'-AMP-activated protein kinase (AMPK) and receptor protein tyrosine kinase (RPTK) pathways

Several recent papers have reported that there is a network of either synergistic or antagonistic cross-talk between 5'-AMP-activated protein kinase (AMPK) and receptor protein tyrosine kinase (RPTK) pathways, e.g. Akt [31,32,37,40], IGF-1 [33], insulin signaling downstream of PKB [36], MEK [34], and c-Raf [43]. Additionally, a cross-talks between AMPK and eukaryotic translation initiation factor 2α (eIF2α) [41] and between c-Raf-1 and Akt [42] were reported. These reports, if confirmed, would suggest that the molecular signaling pathways of how p27 expression might be regulated could be a "tangled web" [42] to say the least.

## Conclusion

Based on the results presented above, we conclude that various nutritional and chemopreventive anti-cancer agents up-regulate expression of p27 in preneoplastic and neoplastic cells, thereby inhibiting G1-to-S phase transition of these cells. (a) These agents appear to up-regulate expression of p27 specifically. (b) Up-regulation of p27 fairly faithfully recapitulates cancer preventive activity of nutritional and chemopreventive anti-cancer agents. (c) Up-regulation of p27 is likely due to the activation of translation rather than transcription of p27.

## Methods

### Reagents

All-*trans*-retinoic acid (atRA), 9-*cis*-retinoic acid (9cRA), 13-*cis*-retinoic acid (13cRA), phorbol 12-myristate 13-acetate (TPA), 1α, 25-dihydroxyvitamin D3 (calcitriol), dexamethasone, 4-hydroxytamoxifen, tamoxifen, 17β-estradiol, genistein, genistin, daidzein, epigallocatechin-3-gallate, epigallocatechin, resveratrol, curcumin, taxifolin, mifepristone (RU486), actinomycin D, anysomycin, Ro-32-0432, hypericin, LY 294002, rapamycin, D-(+)-glucose, and rotenone were obtained from Sigma-Aldrich (St. Louis, MO, USA). ICI 182 780 was purchased from TOCRIS (Ellisville, MO, USA). Triciribine, NSC 119889, AG9, AG18, AG30, AG1024, AG1295, AG1478, AGL2263, PD98059, PD153035, PD169316, SB202190, SB202474, SB203580, IGF-1R inhibitor PPP, ERK activation inhibitor peptide I, ERK activation inhibitor peptide II, COX-1 inhibitor FR122047, COX-2 inhibitor II, salubrinal, compound C, and metformin were obtained from Calbiochem/EMD (San Diego, CA, USA). AICA riboside was purchased from Phoenix Pharmaceuticals, Inc. (Belmont, CA, USA). Dulbecco's Modified Eagle's Medium (DMEM) labeling kit was obtained from Chemicon International (Temecula, CA, USA).

### Cell cultures

Promotion-sensitive (P+, clone 41.5a) JB6 mouse epidermal cell line was kindly provided by Dr. N. H. Colburn (National Cancer Institute, Frederick, MD, USA). JB6 cells were grown in Eagle's Minimum Essential Medium (MEM) supplemented with 5% heat-inactivated fetal bovine serum (FBS), 2% L-glutamine, and antibiotics. Human breast cancer cell lines, MCF7, MDA-MB-231, and AU565, were obtained from the American Type Culture Collection (Rockville, MD, USA). MCF7 cells were grown in Dulbecco's Modified Eagle's Medium (DMEM) containing 4.5 g/L of D-(+)-glucose, supplemented with 10% heat-inactivated FBS, 100 mg/L recombinant human insulin, 2% L-glutamine, and antibiotics. MDA-MB-231 and AU565 cells were grown in the same culture medium without insulin. The incubation was carried out at 37°C in a 5% CO_2 _humidified chamber. All cells were subcultured after trypsinization with 0.05% trypsin-0.02% EDTA solution. The cultures were always maintained below. The cells were checked periodically for mycoplasmal infection by DNA fluorochrome staining.

### Plasmids

Luciferase reporter plasmids containing one of the following proximal 5'-upstream region of the genes were used to transfect the cells: -1745 *cyclin D1 *[2], -963 *cyclin D1 *[2], -963 AP-1mut *cyclin D1 *[2], -8100 *cyclin A *[3], -1797 *p27 *(p27-Kpn I) [4], -774 *p27 *(p27-Apa I) [4], -575 *p27 *(p27-5'UTR) [5,6], -435 *p27 *(p27-MB) [4], -417 *p27 *(p27-IRES) [5,6], and -2320 *p21 *[7]. The control luciferase reporter plasmids that do not contain these inserts were also prepared to test if nutritional and chemopreventive anti-cancer agents were exerting any spurious effects on the backbone rather than the insert of the luciferase reporter plasmids. We found that none of the nutritional and chemopreventive anti-cancer agents tested did not exert spurious effects on the backbone in the human breast cancer cells, but TPA, the three retinoic acids and dexamethasone did stimulate the empty luciferase reporter plasmids in mouse epidermal JB6 cells. Therefore, a proper transfection protocols as described below at the end of the section of Transfection and Luciferase Assay were devised when JB6 cells were exposed to TPA, the three retinoic acids and dexamethasone.

### Transfection and luciferase assay

Transfections were performed according to a published protocol [8] using FuGENE 6 from Roche Applied Science (Indianapolis, IN, USA). In brief, 24 hours before reporter transfection, the cells were seeded into a 60-mm tissue culture dish at a density of 1.5 × 10^5 ^cells/dish and incubated at 37°C in a 5% CO_2 _humidified chamber. Reporter transfection was then carried out with 1 μg of luciferase reporter plasmid and 0.2 μg of pSV-β-galactosidase internal control plasmid (Promega, Madison, WI, USA) mixed with 3 μL of FuGENE 6 solution in 3 mL of FBS-free MEM or DMEM supplemented with only 2% L-glutamine. A minimum of 5-hour incubation at 37°C was needed for transient transfection, followed by 18-hour incubation in either MEM with 5% FBS or DMEM with 10% FBS for recovery. The transfected cells were then starved in either MEM or DMEM with 0.2% FBS for 24 hours. The resulting cells were treated with various compounds in the same culture medium as described in the figure legends. After 24 hours, the treated cells were collected and lysed using Reporter Lysis Buffer (Promega, Madison, WI). The resulting cell lysates were assayed for luciferase activity using Luciferase Assay Kit (Promega, Madison, WI, USA) and TD-20/20 Luminometer (Turner Designs, Sunnyvale, CA, USA). β-Galactosidase activity was measured using chlorophenol red-β-D-galactopyranoside (CPRG) (Sigma-Aldrich, St. Louis, MO, USA) as a substrate.

Each luciferase activity driven by a specific proximal 5'-upstream region of the genes was normalized to β-galactosidase activity, a control for transfection efficiency. Since, depending on the cell types (e.g., JB6), certain nutritional and chemopreventive agents (e.g., the three retinoic acids and dexamethasone) and a tumor promoter (e.g., TPA) stimulated the normalized luciferase activity of empty luciferase reporter vector that do not contain an insert of the specific proximal 5'-upstream region of the genes, the following formula was used in these exceptional cases to correct for this false increase in the relative luciferase activity:

Relative luciferase activity (%) = (Experimental luciferase activity/Control luciferase activity) × 100,

where,

Experimental luciferase activity = {Test compound/None} {Luciferase reporter vector containing a specific promoter insert},

Control luciferase activity = {Test compound/None} {Luciferase reporter vector NOT containing a specific promoter insert}, and

Test compound/None = [Luc(Test)/βGal(Test)]/[Luc(None)/βGal(None)].

### Statistical analysis

An experimental value with statistical significance of P ≤ .05 as compared to control (vehicle alone) by t test is indicated as * on top of the vertical bar.

## Abbreviations

Nonstandard abbreviations: p27, p27Kip1; p21, p21Cip1/Waf1; AMPK, 5'-AMP-activated protein kinase; TSC, tuberous sclerosis complex; mTOR, mammalian target of rapamycin; RPTK, receptor protein tyrosine kinase; PI3K, phosphoinositide 3-kinase; PDK, phosphoinositide-dependent kinase; PKB, protein kinase B; MAPK, mitogen-activated protein kinase; MEK, mitogen-activated protein (MAP) kinase kinase; ERK, ERK MAP kinase; MNK, MAP kinase interacting kinase; m^7^G, 7-methylguanosine; CDK, cyclin-dependent kinase; MNU, *N*-methyl-*N*-nitrosourea; AP-1, activator protein-1; TPA, phorbol 12-myristate 13-acetate; TRE, TPA-response element; atRA, all-*trans*-retinoic acid; 9cRA, 9-*cis*-retinoic acid; 13cRA, 13-*cis*-retinoic acid; N, nucleoside (A, G, U or C); ER, estrogen-receptor; calcitriol, 1α, 25-dihydroxyvitamin D3; RU486, mifepristone; 5'UTR, 5'-untranslated region; IRES, internal ribosome entry site; DMSO, dimethyl sulfoxide; pGL3, pGL3 luciferase reporter vector; SV40, simian virus 40; EGFR, epidermal growth factor receptor; PDGFR, platelet-derived growth factor receptor; IR, insulin receptor; IGR-1R, type 1 insulin-like growth factor receptor; SAPK, stress-activated protein kinase; PKC, protein kinase C; COX, cyclooxygenase; AdoMet or SAM, S-(5'-adenosyl)-L-methionine; eIF2α, eukaryotic translation initiation factor 2α; 4EBP1, eukaryotic translation initiation factor 4E binding protein 1; S6K, p70 S6 kinase; AICA, 5-amino-4-imidazolecarboxamide; Glc, D-(+)-glucose; Met, L-methionine; Cys, L-cysteine; Leu, L-leucine; eIF4E, eukaryotic translation initiation factor 4E; uORF, 5'-upstream open reading frame; PIP_3_, phosphatidylinositol-3,4,5-triphosphate; MEM, Eagle's minimum essential medium; FBS, fetal bovine serum; DMEM, Dulbecco's modified Eagle's medium; EDTA, ethylenediaminetetraacetic acid; CPRG, chlorophenol red-β-D-galactopyranoside; βGal, β-galactosidase; Luc, firefly luciferase.

## Competing interests

The author(s) declare that they have no competing interests.

## Note

Presented in part as a poster at 2005 American Association for Cancer Research/U.S. National Cancer Institute/European Organization for Research and Treatment of Cancer (AACR/NCI/EORTC) International Conference on Molecular Targets and Cancer Therapeutics, November 14–18, 2005, Philadelphia, PA, USA [Eto I: Internal ribosome entry site (IRES) in p27Kip1 mRNA and breast cancer prevention. Programs and Proceedings of the Conference 2005, p.74].
